# Establishment of a Magnetically Controlled Scalable Nerve Injury Model

**DOI:** 10.1002/advs.202405265

**Published:** 2024-09-17

**Authors:** Tuo Yang, Xilin Liu, Rangjuan Cao, Xiongyao Zhou, Weizhen Li, Wenzheng Wu, Wei Yu, Xianyu Zhang, Zhengxiao Guo, Shusen Cui

**Affiliations:** ^1^ Department of Hand and Foot Surgery China‐Japan Union Hospital of Jilin University No.126, Xiantai Street Changchun 130033 China; ^2^ Key Laboratory of Peripheral Nerve Injury and Regeneration of Jilin Province No.126, Xiantai Street Changchun 130033 China; ^3^ School of Mechanical and Aerospace Engineering of Jilin University 5988 Renmin Street Changchun 130025 China; ^4^ Department of Wound Repair, Plastic and Reconstructive Microsurgery China‐Japan Union Hospital of Jilin University No.126, Xiantai Street Changchun 130033 China; ^5^ Department of Chemistry The University of Hong Kong Hong Kong 999077 China

**Keywords:** animal model, magnetic control, neuropathic pain, peripheral nerve injury (PNI)

## Abstract

Animal models of peripheral nerve injury (PNI) serve as the fundamental basis for the investigations of nerve injury, regeneration, and neuropathic pain. The injury properties of such models, including the intensity and duration, significantly influence the subsequent pathological changes, pain development, and therapeutic efficacy. However, precise control over the intensity and duration of nerve injury remains challenging within existing animal models, thereby impeding accurate and comparative assessments of relevant cases. Here, a new model that provides quantitative and off‐body controllable injury properties via a magnetically controlled clamp, is presented. The clamp can be implanted onto the rat sciatic nerve and exert varying degrees of compression under the control of an external magnetic field. It is demonstrated that this model can accurately simulate various degrees of pathology of human patients by adjusting the magnetic control and reveal specific pathological changes resulting from intensity heterogeneity that are challenging to detect previously. The controllability and quantifiability of this model may significantly reduce the uncertainty of central response and inter‐experimenter variability, facilitating precise investigations into nerve injury, regeneration, and pain mechanisms.

## Graphical abstract

This study presents a new animal model of magnetically controlled scalable nerve injury (mSNI), which exerts quantitative and off‐body controllable compression injury to the rat sciatic nerve via a 3D‐printed, implantable, and magnetically controlled clamp. This model can accurately control the injury properties, thus facilitating precise investigations into peripheral nerve injury (PNI), regeneration, and neuropathic pain.

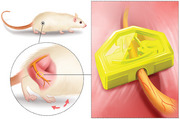

## 1 Introduction

Injuries to the peripheral nervous system (PNS) not only result in the impairment of sensorimotor function, but also continuously generate aberrant peripheral inputs that induce central sensitization and the following chronic neuropathic pain.^[^ ^1^ ^]^ In this process, the injury properties may significantly influence the subsequent pathological changes, nerve regeneration and repair, as well as development and maintenance of neuropathic pain.^[^ ^2^ ^]^ The clinical observations of PNI, particularly in cases of chronic nerve entrapment diseases, are often characterized by the injury properties of progressive nature and intensity heterogeneity. These injury properties significantly influence the prognosis of patient and customization of therapeutic strategy.^[^ ^3^ ^]^ Therefore, it is imperative to establish an animal model that can quantificationally control the injury properties to enable precise investigations in related fields. This is particularly crucial for the understanding of the response of the central nervous system (CNS) to different properties of peripheral inputs, as well as facilitating the development of novel analgesics and therapeutic strategies. However, existing animal models have difficulty controlling injury properties quantitatively, thereby hindering accurate simulation of the pathological processes observed in human patients, particularly central sensitization and remodeling due to varied abnormal peripheral inputs. Furthermore, this also hampers the standardization and comparison of results across different labs. To address such issues, we introduced the concept of off‐body magnetic control and endeavored to develop a novel animal model of magnetically controlled scalable nerve injury (mSNI) that can accurately control the intensity and duration of peripheral nerve constriction injury. With this in mind, we devised a 3D‐printed implant capable of exerting controllable compression on the rat sciatic nerve under the control of an external magnetic field. We demonstrated that the mSNI model can accurately simulate various degrees of chronic and progressive compression injuries observed in human patients by adjusting the magnetic control. Based on this model, we also revealed that the pathological changes did not always exhibit a positive correlation with the intensity of injury; instead, these may manifest specific alterations at certain thresholds, such as variations in axon density and demyelination, thereby highlighting the significance of controlling the injury properties. The development of mSNI model may help facilitate precise investigations into the mechanisms of nerve injury and neuropathic pain, as well as clinical translation of related findings.

## 2 Results

### 2.1 Magnetically Controlled Clamp

Given that the properties of aberrant peripheral inputs induced by PNI are key factors in the development of central sensitization and the following chronic neuropathic pain, the primary objective of our new model was to quantify and standardize such peripheral inputs, thereby facilitating precise investigation of subsequent central activity.^[^ ^4^ ^]^ We hypothesized that the intensity of aberrant peripheral input is positively correlated with the intensity of peripheral nerve stimulation or injury over a large range, so the peripheral input could be quantified and standardized by controlling the intensity of peripheral nerve stimulation or injury. Thus, constriction injury of the peripheral nerve may be the most suitable injury mode for this purpose. To achieve different intensities of compression, we designed an adjustable 3D‐printed clamping apparatus that can be implanted onto the rat sciatic nerve and exert compression of controlled intensity on the nerve trunk. Beyond that, we proposed a solution for a remotely (off‐body) magnetically driven implantable clamp to achieve temporal controllability, avoiding multiple surgeries and minimizing the size of the clamping apparatus and hence the impact on the surrounding tissue.

**Figure 1:**
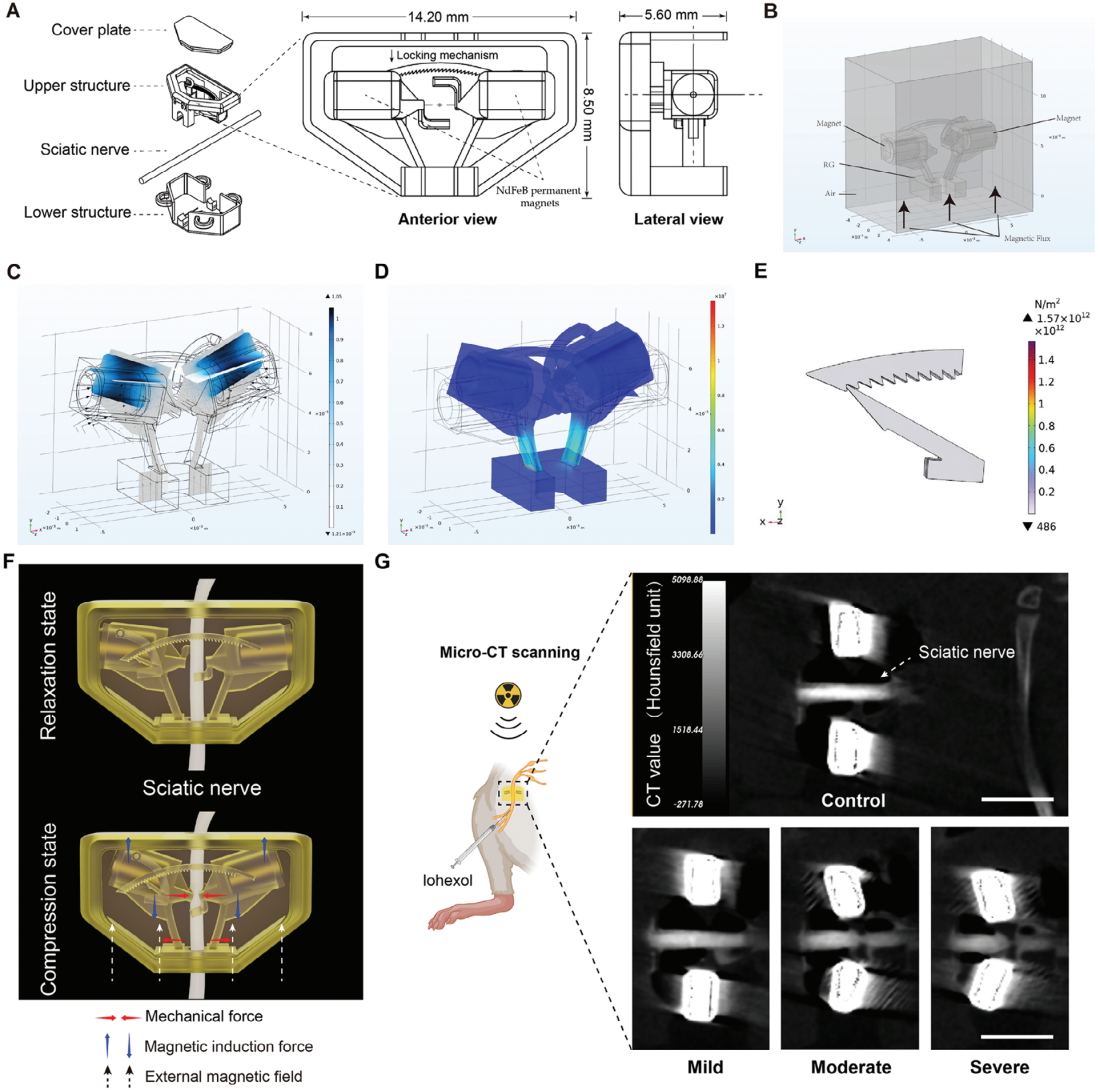
3D‐printed magnetically controlled clamp. A) Clamp mounting illustration and design details of the internal structure of the mClamp. B) Finite element geometry model with permanent magnets, 3D printed RG material, and air. C) Magnetic flux density distribution with 54 mT external magnetic field intensity. D) Stress distribution on a 3D‐printed structure with an external magnetic field of 54 mT. E) Locking mechanism stress distribution at locking state. F) Illustration of the magnetically controlled locking mechanism. The magnetic induction force (blue arrow) exerts a rotational torque on the magnetic beads. With movement along the support direction restricted, the magnetic beads can only generate a lateral tightening force (red arrow) for clamping purposes. The lower side of the support is connected to the bottom, creating a counteracting transverse force (red arrow) that balances out the clamping force. G) In vivo micro‐CT scanning of the compressed sciatic nerve and mClamp under the condition of the final compression intensities of control, mild, moderate, and severe intensities. Scale bar is 5 mm.

After multiple design attempts (Supporting Information), we selected the optimal design to implement the adjustable clamping mechanism by embedding NdFeB magnetic beads within a 3D‐printed polymer (light‐curing composite resins) structure (**Figure** **1**A). A channel, of a larger diameter than a typical rat sciatic nerve trunk, was designed for the passage of the nerve trunk through the device. It can also serve as a conduit for introducing biomaterials such as hydrogels into the interior of the device, enabling interventions at the injury site in related studies. Here, hyaluronic acid was injected to prevent local tissue scarring and hyperplasia. Thus, we designed and manufactured a magnetically controlled clamp (mClamp) apparatus that can be implanted on the rat sciatic nerve to produce a locking effect under the control of an external magnetic field (Figure S1A,B, Supporting Information). This mClamp can exert a pre‐defined intensity and duration of compression on the rat sciatic nerve for specific studies.

### 2.2 Finite Element Simulation and In Vitro Test

The performance of the mClamp under magnetic forces was simulated and evaluated by finite element method (FEM) simulations for the optimization of the design and fabrication.^[^ ^5^ ^]^

We employed coupled multiphysics field simulations to track the deformation of magnetically controlled materials, especially the movement of the champ tips, driven by the magnetic force induced from the beads.^[^ ^6^ ^]^ Due considerations were given to the magnetic field and the mechanical properties of the materials (Figure 1B; Figure S1C, Supporting Information). Then we analyzed the cloud diagram representing the magnetic flux distribution across the mid‐axis cross section (Figure 1C; Figure S1D–F, Supporting Information).^[^ ^7^ ^]^ We also calculated the stress distribution for the clamp with the structure cured from an RG resin (details in Table S1, Supporting Information), to tailor the design configuration (Figure 1D; Figure S1G–I, Supporting Information). These simulations evaluated the induced magnetic force and the stress on the supporting beam, enabling us to minimize the device size without material failure.

The primary purpose of the locking mechanism was to maintain the clamp's position of compression after removal of the external magnetic field. The process under consideration was simulated using a finite element model, which was subsequently employed to analyze the pushing force needed by the “rack” for the locking process (Figure 1E; Figure S2A,B, Supporting Information). The maximum force that can be created to compress the nerve is equal to the driving force produced by the permanent magnet in reaction to the external magnetic field minus the elastic resistance for the rack to pass the locking teeth. The force necessary to overcome the elastic resistance of the locking teeth was calculated as 0.012 N (Figure S2C, Supporting Information) while the permanent magnet can produce a maximum push force of 0.12 N. Thus, a remaining force of 0.108 N is available to compress the nerve, which is sufficient.

Before applying it in rats, we tested the mClamp in vitro to determine whether it could exert controlled degrees of compression on a simulated nerve trunk under an external magnetic field. A miniature camera with a macro lens support was placed inside the coil of the magnetron drive unit to facilitate the in vitro test and provide real‐time monitoring of the clamp's performance. The external magnetic field induced a rotational torque on the magnetic beads, which were constrained by the support and can only generate transverse force toward the center. This clamping effect can be adjusted to different gears through the locking mechanism, resulting in varying degrees of compression on the simulated nerve (Figure 1F; Figure S2D and Video S1, Supporting Information). Since gear less than 5 did not exert any pressure on nerve, we identified gear 5 to 12 as an optimal range for compression. We also found that there were some variations in the initial clamping force. This may be because additional supports were added for the unique structures during the 3D printing process, the structures were likely to change if these supports were removed, and the printed material's degree of cross‐linking and curing was not uniform. To ensure standardization and reproducibility of our study, each mClamp was tested to determine the initial strength of the magnetic field needed for its clamping, as well as the corresponding voltage and current in the coil. The mClamps that did not meet the design criteria were excluded from further in vivo studies.

### 2.3 mClamp Exerted Spatiotemporally Controllable Compression

After confirming that the mClamp can produce varying degrees of locking under magnetic control in vitro, we then evaluated the performance of the mClamp in vivo. We first employed micro‐CT scanning to visualize the compression exerted by the mClamp onto the sciatic nerve. The mClamp was implanted onto the sciatic nerve of rats and locked to different degrees under the control of an external magnetic field. Subsequently, micro‐CT scanning was conducted to assess the extent of compression on the nerve truck. The CT‐reconstructed images showed that the mClamp worked well in vivo and could exert different intensities of compression to the rat sciatic nerve (Figure 1G).

**Figure 2:**
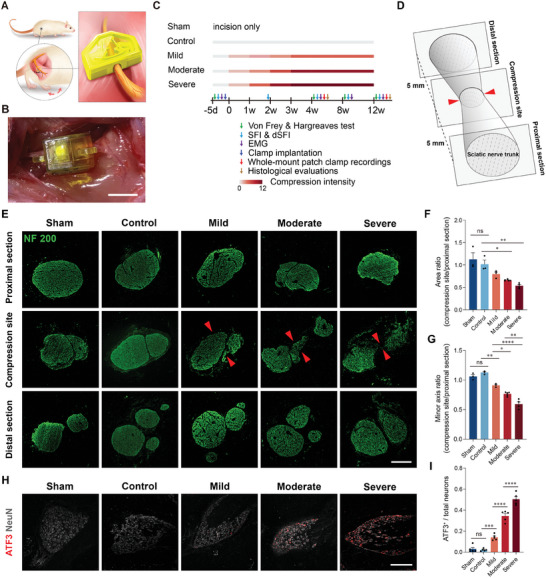
mClamp exerted spatiotemporal controllable compression to the rat sciatic nerve. A) Schematic of experimental design for implantation of the mClamp. B) Exterior of the implanted mClamp on rat sciatic nerve. Scale bar is 2 mm. C) Grouping and timeline. D) Schematic for the evaluation of the area of different sections of the sciatic nerve. Compression points are indicated by red arrowheads. E–G) Immunostaining of NF 200 for different sections of sciatic nerve trucks (E) and their quantification (F,G) at 12 weeks after compression. Compression points are indicated by red arrowheads. Scale bar is 50 µm. *n* = 3, 3, 3, 5, and 4 rats for the sham, control, mild, moderate, and severe group. H,I) Immunostaining of ATF3 in affected DRGs (H) and their quantification (I) at 12 weeks after compression. Scale bar is 500 µm. *n* = 5 rats per group. All data are expressed as the mean ± s.e.m. Statistical comparisons were conducted with one‐way ANOVA followed by Bonferroni's post hoc test.

Based on the above, we then aimed to determine quantitatively whether the mClamp could induce controllable intensities of compression onto the sciatic nerve. As human patients with nerve entrapment diseases often exhibit pathological features that are difficult to simulate using existing animal models, including varying compression intensities, varying durations, and progressive compression, we were also interested in whether the spatiotemporal off‐body controllability of the mSNI model could help simulate these features and provide controllable peripheral input. The following experimental design was devised for these purposes (**Figure** **2**A–C).

First, to determine whether the mSNI model could be used to cause graded nerve compression, rats were randomly divided into five groups, including the sham (incision and nerve exposure performed without mClamp implantation), control (mClamp implantation without compression), mild, moderate, and severe groups. Second, in the absence of therapeutic intervention, the pathological process of human peripheral nerve entrapment diseases tends to be gradually aggravated and then remains stable for a long time, with less spontaneous remission.^[^ ^8^ ^]^ To simulate this chronic and progressive feature, compression of the mClamp in the mild, moderate, and severe groups was increased in stages by control of the external magnific field once a week until the maximum intensity of compression was reached in the third week (we defined 0 d when the compression began, Figure S2E, Supporting Information). Finally, considering the prolonged course of nerve entrapment diseases in human patients, our study primarily focused on two pivotal time points: the culmination of the gradual compression process (4 w) and the chronic phase (12 w), aiming to elucidate the pathological progression of the mSNI model at different stages of chronic compression.

To evaluate the physical compression of the sciatic nerve by the mClamp, sciatic nerve cross‐sectional samples from mSNI rats were assessed at 12 w (Figure 2D,E), particularly at the compression site (7 mm proximal to the bifurcation of the sciatic nerve in the sham group) (Figure 2F). The proximal segment of the injury site served as a control, ensuring elimination of any inter‐rat variations in the nerve trunk cross‐sectional area. Considering that the mClamp primarily applied compression in the direction perpendicular to the long axis of the sciatic nerve trunk, we also assessed the ratio of the minor axis (compression site to proximal section, Figure 2G). The data showed that, compared to the proximal section, the area and minor axis of the compression site exhibited no significant difference in the sham and control groups. However, in the mild, moderate, and severe groups, the sciatic nerve trunks showed compression related to the degree of mClamp locking. These results indicated that the compression on the sciatic nerve trunk from the mClamp was controllable and quantifiable and that the implantation of the mClamp (without locking) did not cause compression of the nerve trunk.

To determine whether such controllable compression could induce corresponding degrees of axonal damage, we performed immunostaining of ATF3, a marker of injured/axotomized neurons, in the affected dorsal root ganglions (DRGs). We found that the affected DRG neurons exhibited varying degrees of up‐regulation of ATF3 expression corresponding to their compression intensities, with significant differences among different compression groups (Figure 2H,I), indicating that the mClamp induced controllable degrees of axonal damage.

### 2.4 mSNI Induced Controllable Hyperpathia

**Figure 3:**
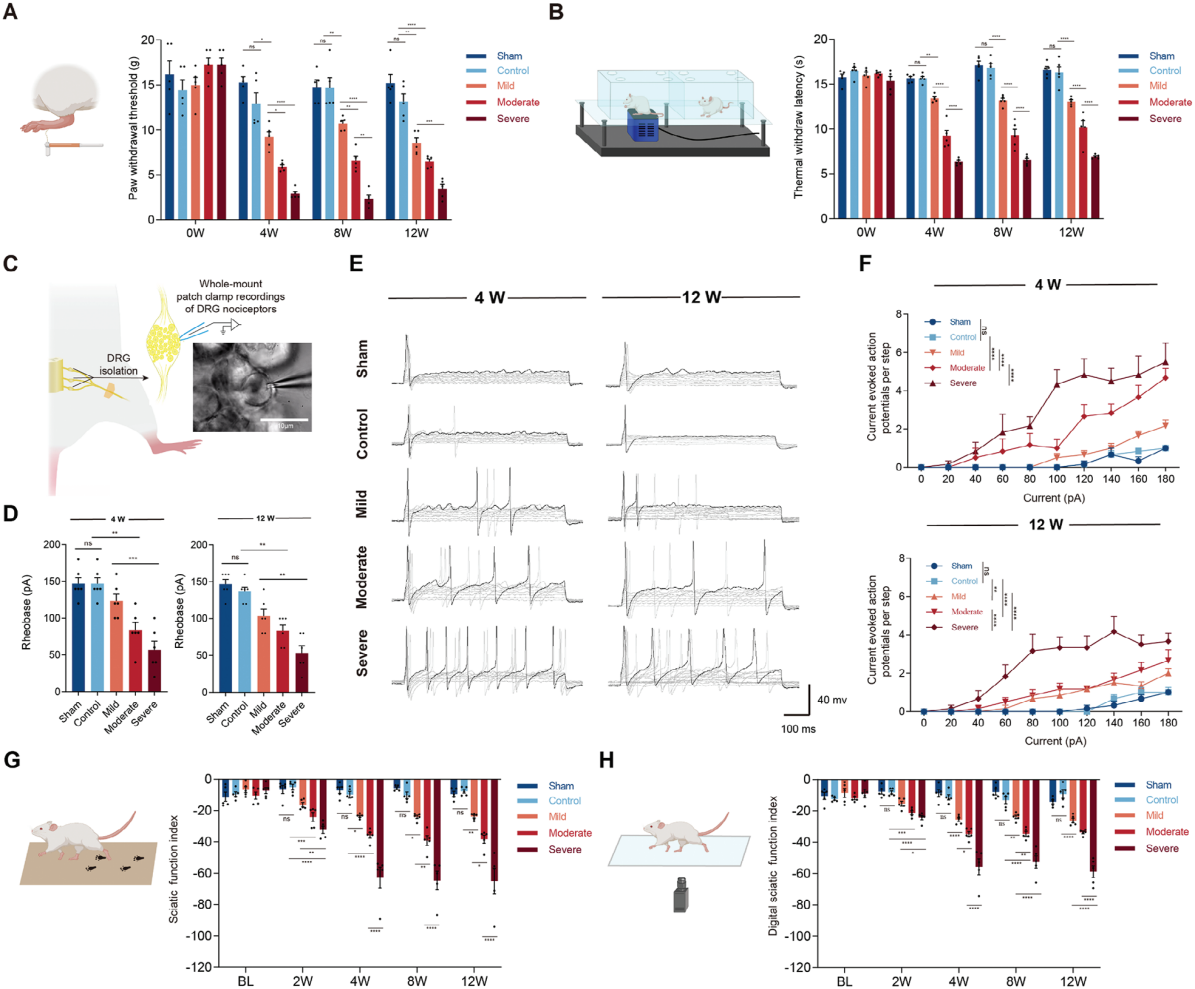
mSNI induced controllable nociceptor hypersensitivity, motor and sensory dysfunction. A,B) mSNI rats exhibited graded mechanical (A) and thermal (B) pain hypersensitivity. *n* = 5 rats per group. C) Schematic of experimental design for whole‐mount patch clamp recordings of rat DRG and a small‐diameter DRG neuron with attached recording pipette under infrared phase contrast microscope. D) Quantification of rheobase. E,F) Representative traces (E) and quantification (F) of current‐evoked action potentials. *n* = 6 neurons from at least three separate rats (D,F). G,H) mSNI rats exhibited graded motor dysfunction in SFI (G) and dSFI (H) evaluations. *n* = 5 rats per group. All data are expressed as the mean ± s.e.m. Statistical comparisons were conducted with two‐way ANOVA followed by Bonferroni's post hoc test (A,B,F,G,H) or one‐way ANOVA followed by Bonferroni's post hoc test (D).

As mClamp can exert controllable compression on the sciatic nerve, our interest was then focused on whether this controllable compression could induce corresponding levels of sensorimotor dysfunction and peripheral inputs. To address this question, we evaluated mSNI‐induced hyperpathia. Mechanical and thermal hyperpathia were detected by the Von Frey and Hargreaves methods, respectively. We found that from 4 weeks after the start of the compression, the experimental rats showed graded mechanical and thermal hyperalgesia corresponding to the compression intensity (**Figure** **3**A,B). This graded hyperalgesia continued to the maximum observation period (12 weeks after the start of the compression) and remained stable, with a significant difference. Notably, no spontaneous relief from hyperalgesia in the mild, moderate, and severe groups was observed within the observation time, which is quite different from the traditional chronic constriction injury (CCI) model.^[^ ^9^ ^]^ In addition, there was no significant difference between the sham and control groups in hyperalgesia within the observation time, indicating that long‐term implantation of the mClamp caused limited chronic inflammation and scar hyperplasia, which were insufficient to cause significant changes in nociception.

### 2.5 mSNI Induced Controllable Nociceptor Hypersensitivity

Although the above pain behavioral tests partially reflected the mSNI‐induced aberrant peripheral inputs, central components still cannot be ruled out. To accurately assess aberrant peripheral inputs, we evaluated DRG nociceptor hypersensitivity in mSNI rats.^[^ ^1^, ^10^ ^]^ Whole‐mount patch clamp recordings of ipsilateral DRG small‐diameter (<25 µm) nociceptive neurons were employed to examine mSNI‐induced variation in nociceptor excitability ex vivo (Figure 3C). At 4 weeks after the start of the compression, the current‐evoked action potentials and rheobase of nociceptors in the sham group exhibited no significant difference from those in the control group, indicating that implantation of the mClamp caused limited collateral damage and inflammation (Figure 3D–F). On the other hand, DRG nociceptors in the mild, moderate, and severe groups showed graded hypersensitivity related to the compression degree, indicating that mSNI could induce a controllable intensity of nociceptor hypersensitivity. We also checked nociceptor excitability 12 weeks after the initiation of the compression. The excitability of DRG nociceptors in the control group was not significantly different from that of DRG nociceptors in the sham group, indicating that long‐term implantation of the mClamp caused a limited chronic inflammatory response and scar hyperplasia, which was insufficient to affect nociceptor sensitivity. Although the nociceptors subjected to 12 weeks of compression exhibited lower excitability than those subjected to 4 weeks, rats subjected to different compression intensities still showed gradations in the frequency of current‐evoked action potentials and the rheobase. The above observations are consistent with the results of pain behavioral studies and demonstrate that the mSNI model could induce peripheral input of controllable intensity over a long‐time span.

### 2.6 mSNI Induced Controllable Motor Dysfunction

It is noted that motor function deficiency reflects the intensity of nerve injury and the development of novel analgesics requires elimination of any potential influence on motor function recovery. Therefore, we characterized the motor function of mSNI rats using the sciatic nerve function index (SFI, Figure 3G). The data showed no significant difference in the SFI between rats in the control and sham groups throughout the observation period. On the other hand, the rats in the mild, moderate, and severe groups showed varied degrees of motor dysfunction corresponding to the intensity of compression and did not show a recovery tendency within 12 weeks of observation. We further employed the DigiGait system (Boston, MA) to evaluate the digital sciatic nerve function index (dSFI) and found that the results were basically consistent with the SFI results (Figure 3H; Figure S3A, Supporting Information). In addition, we also evaluated other indicators of motor function, including myodynamia and electromyographic signals of the affected tibialis anterior (TA) muscle, and these results were also consistent with those of the SFI (Figure S3B–E, Supporting Information). Collectively, these data indicate that 1) the mClamp implantation did not impair motor function; 2) the mClamp could exert controllable compression on the sciatic nerve, thus inducing graded motor function deficiency related to the compression intensity; and 3) the motor function deficiency induced by the mSNI model remained stable for a long time after modeling, without spontaneous recovery during the observation period.

### 2.7 mSNI Simulated the Progression and Recovery of Chronic Nerve Entrapment Syndromes

Since the nerve trunk swelling induced by ligations may contribute to the early development of neuropathic symptoms in CCI model, we next characterized the progression of motor function deficiency and pain hypersensitivity of mSNI rats at early stage and determined whether the nerve swelling played a role in the early symptoms of mSNI. We found no statistical differences between the groups prior to the first pressurization (7d), whereas differences between different compression intensities gradually became apparent between 14 and 21 days (Figure S4A–D, Supporting Information). This may be due to our experimental design of a chronic progressive compression process, where local pressure remained minimal during the early stage (<7d), resulting in insufficient nerve swelling to induce notable neurological symptoms. Such chronic and progressive compression, rather than acute tight ligations causing severe edema, may more closely resemble the pathology observed in human patients with chronic nerve entrapment disorders.^[^ ^3a^ ^]^

The efficacy of surgical decompression has always been one of the controversial topics in the field of related diseases.^[^ ^11^ ^]^ To simulate such clinical situations and characterize the recovery following surgical decompression of varying intensities of nerve compression, we removed the mClamp at day 28 and evaluated the neuropathic symptoms in mSNI rats (Figure S4E–H, Supporting Information). We found that rats in the mild group almost completely recovered within 3 weeks, whereas those in the severe group exhibited minimal indications of recovery. Interestingly, for the rats suffered from moderate compression, motor function was recovered at 3 weeks compared with that before decompression (Figure S4E,F, Supporting Information) while no notable improvement in pain hypersensitivity was detected (Figure S4G,H, Supporting Information), indicating a significantly enhanced central sensitization compared with the control group.

### 2.8 mSNI Induced Controllable Histopathological Changes

From the above, we demonstrated that the mSNI model could provide relatively controllable injury properties as well as peripheral inputs by accurately exerting sciatic nerve compression. The subsequent focus of our investigation was to ascertain the presence of concomitant histopathological alterations associated with such graded injury intensities.

**Figure 4:**
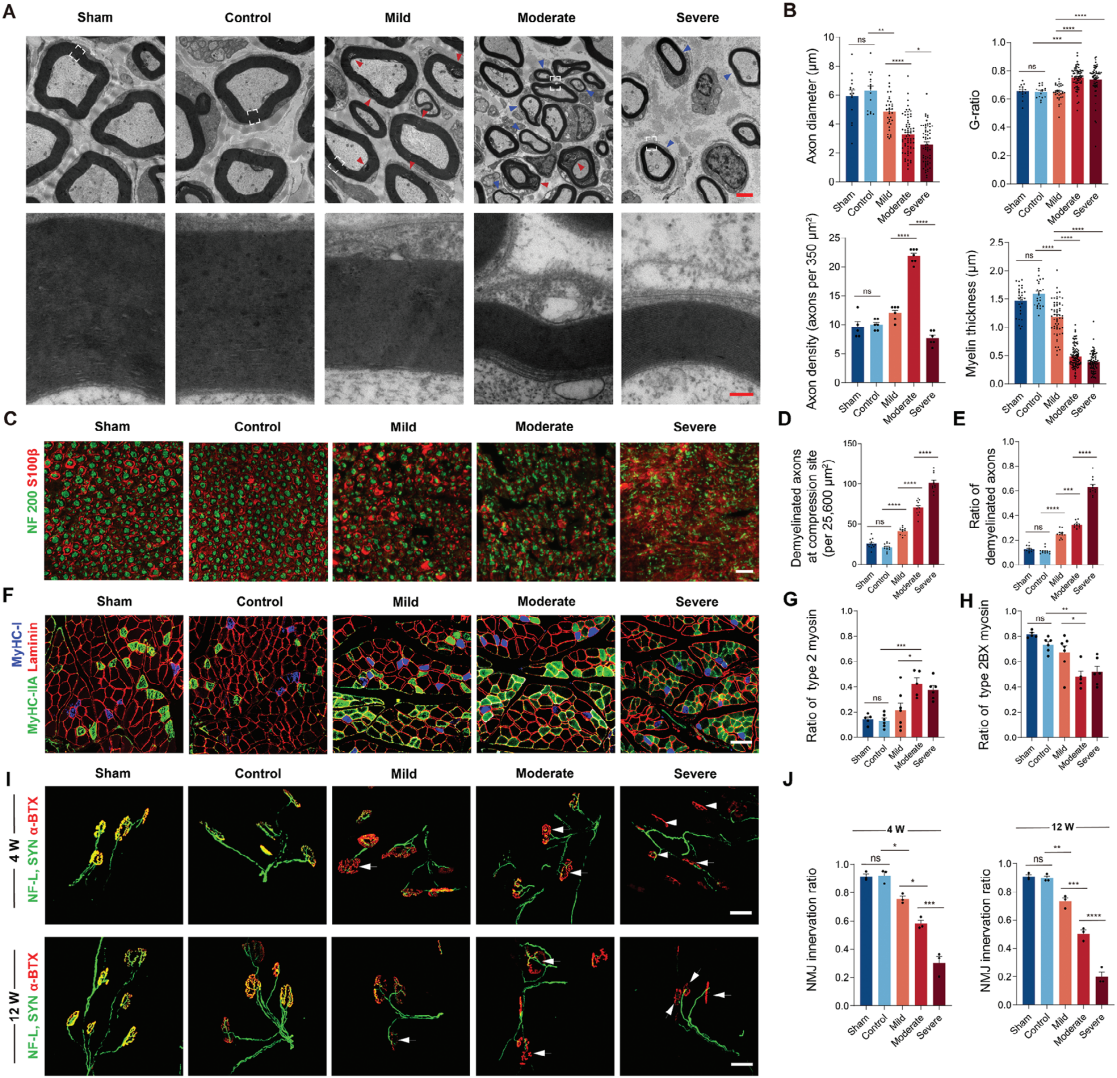
mSNI induced controllable histopathological changes. A,B) Electron micrographs (A) and their quantification (B) of the cross section at 5 mm distal to the injury site 12 weeks after compression. Areas of degeneration as well as axons in demyelination are indicated by red arrowheads. Axons in remyelination are indicated by blue arrowheads. Scale bar is 2 µm (upper) and 200 nm (lower). For axon diameter and G‐ratio, *n* = 15, 16, 33, 68, and 61 axons from 5, 6, 6, 7, and 6 rats. For axon density, *n* = 5, 6, 6, 7, and 6 rats. For myelin thickness, *n* = 28, 26, 60, 100, and 77 axons from 5, 6, 6, 7, and 6 rats. C) Immunostaining of the axon (NF 200, green) and myelin sheath (S100β, red) of the injured sciatic nerve 12 weeks after compression. Scale bar is 20 µm. D,E) Quantification of demyelinated axons at compression site (D) and ratio of demyelinated axons (E). *n* = 4 rats per group. F) Immunostaining of the myosin, including Laminin (red), myosin type 1 (MyHC‐1, blue), and myosin type 2A (MyHC‐IIA, green). Scale bar is 100 µm. G,H) Quantification of type 2 (G) and type 2BX (H) myosin. *n* = 5, 6, 7, 5, and 6 rats. I,J) Immunostaining for NMJ (I) and their quantification (J). Nerve fiber and presynaptic membrane (NF‐L and SYN, green), postsynaptic membrane (α‐BTX, red). Scale bar is 50 µm (I). *n* = 3 rats per group. All data are expressed as the mean ± s.e.m. Statistical comparisons were conducted with one‐way ANOVA followed by Bonferroni's post hoc test.

The initial emphasis was placed on the pathological alterations in myelin and axons in mSNI rats. The axon diameter, G ratio (axon diameter/axon + myelin diameter), axon density, and myelin thickness at the injury site of the sciatic nerve were evaluated under electron microscopy 12 weeks after compression began (**Figure** **4**A,B). The results revealed a reduction in the axon diameter and myelin sheath thickness, accompanied by an increase in the G ratio, with increasing compression intensity. This observation aligned with our initial expectations and suggests a direct correlation between the intensity of compression and the extent of demyelination of the affected nerve. Interestingly, we observed a significant increase in the myelin density in mSNI rats subjected to moderate compression compared to mild compression. However, with a further increase in the compression intensity, there was a sudden decrease in the axon density in the severe group. The histological findings revealed extensive degeneration of the myelin sheath in the mild group, indicating a preliminary state of demyelination (Figure 4A, red arrow). In the moderate group, the axons exhibited high density and signs of both degeneration and regeneration, with a large number of axons undergoing remyelination (Figure 4A, blue arrow) alongside a small number of degenerating axons. However, among the sciatic nerves subjected to severe compression, few axons were observed; considering that the diameter of the sciatic nerve in the severe group was also significantly reduced (Figure 2E,F), the total number of remaining axons was minimal. These results highlight distinct pathological changes resulting from varying compression intensities and may explain the clinical observation that patients with chronic nerve entrapment diseases tend to exhibit spontaneous recovery in mild to moderate cases but complete inactivation in severe cases – a phenomenon that is challenging to replicate using traditional models.^[^ ^8^ ^]^ Similarly, immunostaining of the axon and myelin sheath of the injured sciatic nerve after 12 weeks of compression showed graded demyelination correlated with the compression intensity, indicating that compression intensity may greatly influence demyelination‐related pathological changes (Figure 4C–E; Figure S5A, Supporting Information).

We then evaluated the tissue inflammation of affected nerves through H&E staining at 4th week (Figure S5B,C, Supporting Information). Immune infiltration was observed gradually increased from the mild to moderate and severe groups, indicating that the mSNI model induced varying degrees of tissue inflammation accordingly. Although the control group exhibited mild tissue edema due to mClamp implantation compared with the sham group, there was no significant difference in terms of immune infiltration.

We next explored mSNI‐induced pathological changes in muscle‐related tissues. Chronic denervation leads to changes in the myosin ratio of the innervated muscle, which reflects the degree of pathological changes. Myosin immunostaining of the affected TA muscle after 12 weeks of chronic compression suggested that chronic entrapment injury of the peripheral nerve led to an increase in the proportion of type 2 myosin in the innervated muscle, especially in cases of moderate and severe compression (Figure 4F–H; Figure S5D, Supporting Information). Given that PNI results in denervation of neuromuscular junctions (NMJs) and atrophy of the innervating muscles,^[^ ^12^ ^]^ we then evaluated NMJ denervation (Figure 4I,J), the wet‐weight ratio of the TA and gastrocnemius (GA) muscles, the muscle fiber cross‐sectional area, the muscle fiber diameter, and the proportion of collagen fibers to muscle cross‐sectional area (Figure S6A–H, Supporting Information). We found that mSNI rats exhibited graded pathological changes in the innervating muscles according to the intensity of nerve compression. These results indicated that 1) the pathological changes in innervating muscles were positively correlated with the intensity of compression; 2) mSNI could induce a controllable degree of pathological changes in innervating muscles; and 3) the pathological changes in innervating muscles induced by different compression intensities were significantly different, implying the importance of controlling and standardizing compression intensity in relevant preclinical studies.

## 3 Discussion

Articles related to the most commonly used models of neuropathic pain, including CCI, spinal nerve ligation, and spared nerve injury (SNI), have been cited more than 10 000 times in total, and articles related to CCI, a representative model of chronic nerve constriction injury, have been cited more than 5 000 times (data from September 2023, Web of Science), indicating a great need for such animal models. The main features of these models include a short modeling time, simple operation, and one‐time modeling.^[^ ^9^, ^13^ ^]^ Many achievements have been made in this field based on these animal models, which have greatly improved the knowledge of neuropathic pain.^[^ ^14^ ^]^ However, preclinical studies based on these models also face some difficulties, thus achieving further developments in this field difficult.^[^ ^15^ ^]^ The primary challenge lies in the inability of current models to accurately replicate the pathological features observed in human patients, especially the intensity and duration heterogeneity.

Our study demonstrated that the intensity heterogeneity of nerve compression may result in quite different peripheral inputs. We also observed robust regenerating fibers when the compression intensity was changed from mild to moderate, but reverted to an inactive state when the compression intensity was further increased to severe. These specific pathological changes caused by intensity heterogeneity have often been overlooked, but they may play a crucial role in the development of chronic pain and nerve regeneration. On the other hand, existing commonly used models induced neuropathic pain peaks within several days and exhibits rapid spontaneous recovery (except SNI).^[^ ^16^ ^]^ These models are oversimplified in time scale (although in rodents) since chronic pain is defined as pain that lasts more than 3 months and rarely recovers spontaneously.^[^ ^17^ ^]^ As emerging studies have begun to focus on the time‐dependent mechanisms in pain,^[^ ^18^ ^]^ existing time‐reduced models are difficult to meet the growing demand. In the present study, we set a prolonged time scale that is quite different from those of existing models, and demonstrated that the chronic inflammation and scar hyperplasia caused by long‐term mClamp implantation were limited, and the mSNI rats exhibited ideal pathological progression without spontaneous recovery over a long time span. By this way, mSNI model may help replicate the intensity and duration heterogeneity in clinical observations that were difficult to replicate previously as well as reveal some intensity‐dependent or time‐dependent mechanisms in nerve injury, regeneration, and neuropathic pain.

The progressive compression resulting from inflammation and fibrosis surrounding the injured nerve is a significant pathological characteristic observed in human patients with nerve entrapment diseases, which have a potential influence on injury progression and pain development.^[^ ^19^ ^]^ However, such progressive compression‐induced pathological changes have been rarely reported in preclinical studies. In the present study, we sought to characterize this pathological progression through multiple attempts. We selected two key time points to demonstrate the mSNI‐induced progressive compression and the following pathological alterations, 4 weeks of compression, when the pressurization was just finished, and 12 weeks of compression, in the chronic stage. We also profiled the early progression of the motor function deficiency and neuropathic pain during the increasement of compression. Additionally, in most studies, young mice (mostly 4–6 weeks old) are used for patch clamp recordings due to the poor cell tolerance, high reactive oxygen species generation, and low membrane flexibility of older neurons.^[^ ^10^, ^20^ ^]^ However, considering that our study involved a prolonged time course of chronic compression and that older individuals are likely to be affected by chronic pain,^[^ ^21^ ^]^ we employed whole‐mount DRG patch clamp recordings to assess mSNI‐induced nociceptor hypersensitivity, thus providing rare data from older rats revealing electrophysiological features of DRG nociceptors suffering from prolonged progressive constriction injury.

The relationship and interactions between peripheral and central circuit are key points in studying neurological mechanisms, especially pain mechanisms.^[^ ^2^, ^22^ ^]^ However, the insufficient capability to accurately manipulate peripheral inputs makes studies on the central circuit being confined to the CNS and less closely related to peripheral elements. Some scholars have used peripheral blocking to control peripheral input.^[^ ^23^ ^]^ However, current interventions on peripheral input, such as lidocaine and tetrodotoxin, are more likely to act in an all‐or‐nothing manner, lacking controllability and quantifiability. The mSNI models offer a promising solution to this puzzle by providing us with a precise and quantitative means to manipulate peripheral inputs. Furthermore, this precise manipulation induced by the mechanical device may mitigate the variability introduced by manual ligation, which is one of the challenges encountered in traditional models such as the CCI model.^[^ ^9^, ^16^, ^24^ ^]^

## 4 Conclusion

Overall, the newly developed mSNI model overcomes several key issues for further research in related fields and potentially promotes clinical translation. Moreover, we introduced the concept of off‐body magnetic control to the field of disease modeling. This advancement significantly enhances the maneuverability and accuracy, and demonstrates the potential for applications in other animal models and even human patients, enabling precise manipulation of pathological processes within organisms. Future development may focus on the following aspects to improve the mSNI model. First, the present study primarily focused on mSNI‐induced peripheral inputs and indicators within the PNS, and it is important to explore further the response, sensitization, and remodeling of the CNS caused by diverse mSNI‐induced peripheral inputs. Second, this study solely examined the mClamp performance in rats, and the material can be tailored to enable its utilization in mice, large mammals, and even primates. Finally, it is necessary to streamline and standardize the production and operation protocols of both the mClamp and the magnetic control unit, to minimize the cost of utilization, facilitate their commercialization and widespread adoption among laboratories, and ultimately benefit the patients with PNI, chronic nerve entrapment diseases, and chronic neuropathic pain.

## 5 Experimental Section

### Structural Design and FEM

The mClamp 3D structural model was designed using Solidworks (Dassault Systemes, China). After the structural design of the mClamp was finalized, the geometry file was exported to COMSOL software (COMSOL, China) for FEM simulations.^[^ ^25^ ^]^ The simulated results were analyzed to optimize the structural design.

### 3D Printing and Fabrication

The optimized mClamp design was exported to STL file for 3D printing. The parts were fabricated using MicroArch S240 (BMF, China) 3D printer. A pair of cylindrical N52 NdFeB permanent magnets, each measuring 2 mm in diameter and 3 mm in length, were mounted into the mClamp with opposite magnetic polarization directions using a bio‐adhesive (LocTITE 4011).

### Magnetic Control Unit

A magnetic control unit was in‐house built utilizing a spiral coil of 3800 windings to generate a steady and controllable magnetic field. The inner diameter of the coil was 120 mm. Two sets of three‐axis translational moving platforms with aluminum brackets were fitted to adjust the positions regarding to the coil. The magnetometer and the miniature camera with macro lens were attached on one of the brackets. The other one was used for the in vitro test. The maximum power of the power supply was 8 kW with adjustable output voltage ranging from 0 to 800 VDC. The coil was enclosed in an aluminum alloy case in order to incorporate a liquid cooling system with to dissipate the heat generated by the coil. The thermometer and the ohmmeter were installed to monitor the coil temperature and resistance.

### In Vitro Test

The fabricated mClamps assembled with permanent magnets were attached onto the bracket of the translational moving platform and placed in the center of the magnetic field generated by the coil. The miniature camera was placed aside of the mClamp to record its movement. The next step was to adjust the voltage of the power supply to a required value while the coil was isolated from the power supply. Connecting the coil with the power supply for continuous 5 s and switch off the power supply was able to generate the controllable intensity of the magnetic field to drive the mClamp. After removing the mClamp from the bracket, it was placed under a microscope to observe the closure of the mClamp for evaluation. The degrees of closure of the mClamp and its corresponding voltage and current were recorded. The above steps were repeated with different levels of voltage for each mClamp to record the closure‐current relationship. After the tests, the locking was released to allow the mClamp to recover its closure status.

### Animals

Male adult Sprague Dawley rats were purchased from Changsheng Biotechnology (China). All animal experiments were carried out according to protocols approved by the Animal Ethics and Welfare Committee of Jilin University. In compliance with the requirements of Jilin University and the state for the ethical welfare of experimental animals, the feeding conditions were carried out in strict accordance with Laboratory animal Requirements of environment and housing facilities of China (GB14925), and the experimental animals were free to drink and eat. Rats were randomly divided into five groups named: sham, control, mild, moderate, and severe.

### mClamp Implantation

Rats of 10‐week‐old (220–250 g) were anesthetized with isoflurane by an animal anesthesia apparatus (RWD, China). After disinfecting the right hind limb, an incision was made at the femoral projection to expose the right sciatic nerve. The mClamp was meticulously positioned on the sciatic nerve trunk. To prevent chronic scar hyperplasia after long‐term implantation, hydrogel was injected (hyaluronic acid + rapamycin) onto the surface of mClamp. After the incision was closed, carprofen (5 mg k^−1^g per 12 h) was administered subcutaneously for 3 consecutive days to provide postoperative analgesia.

### Micro‐CT Scanning

To visualize the compression exerted by the mClamp onto the sciatic nerve in vivo, 0.05 mL of iohexol was injected as a contrast medium into the rat sciatic nerve with mClamp implantation. The rats were placed in the micro‐CT (NMC‐200, PINGSENG Healthcare, China) sample chamber, with a tube voltage of 90 kV and a tube current of 40uA. The regions of interest for scanning were chosen to be the areas surrounding the femur and knee joint of the rats, followed by image reconstruction using the iterative method on the Avatar software (Version 1.7.0, PINGSENG Healthcare, China).

### Reagents and Antibodies

Chemicals, unless other indicated, were purchased from Sigma‐Aldrich. The information of primary antibodies used was as follows: anti‐neurofilament (NF‐L, rabbit, 1:300, Cell Signalling Technology, Cat# 2837), anti‐synaptophysin (SYN, rabbit, 1:800, Cell Signalling Technolog, Cat# 5297), CF568‐labeled α‐bungarotoxin (α‐BTX, Biotium, 1:2000, Cat# 00006), anti‐neurofilament 200 (NF‐200, mouse, 1:80, Cat# N5389), anti‐S100 beta (S100β, rabbit, abcam, 1:500, Cat# ab52642), anti‐myosin heavy chain types 1(MyHC‐1, mouse, 1:75, DSHB, Cat# AB2235587), anti‐myosin heavy chain types 2a (MyHC‐2a, SC‐71, 1:150; DSHB, Cat# AB2147165), anti‐laminin (laminin, rabbit, abcam, 1:100, Cat# ab11575), anti‐ATF3 (ATF3, rabbit, abcam, 1:400, Cat# ab207434), anti‐NeuN (NeuN, chicken, Aveslab, 1:500, Cat# NUN). The information of secondary antibodies used was as follows: donkey anti‐mouse IgG; Alexa Fluor 488, 1:500, abcam, Cat# ab150113; donkey anti‐rabbit IgG; Alexa Fluor 546, 1:500, abcam, Cat# ab2534016; goat anti‐rabbit IgG, Alexa Fluor 488, 1:500, abcam, Cat#ab150077; goat anti‐mouse IgG2b, Alexa Fluor 647, 1:500, Invitrogen, Cat# A‐21245; goat anti‐mouse IgG, Alexa Fluor 488, 1:500, Invitrogen, Cat# A‐21121.

### Muscle Wet Weight Assessment

After anesthetizing the rats with isoflurane, the anterior tibial muscles from both ipsilateral and contralateral sides were carefully isolated and promptly transferred to an electronic balance (Mettler‐Toledo, USA) for precise measurement. Rats were then deeply anesthetized with and perfused for further studies. The wet‐weight ratio was determined by the wet‐weight of ipsilateral muscle / the wet weight of contralateral muscle.

### Immunofluorescence

For nerve cross‐section staining, the sciatic nerve samples were dehydrated in 30% sucrose at 4 °C for 48 h. The transverse sections (12 µm) of proximal section, compression site, and distal section were obtained using a cryostat microtome (CM1950, Leica, Germany). The Sections were rinsed with PBS and incubated with blocking buffer (1% Triton X‐100 in 3% BSA and 2% goat serum) for 2 h at room temperature and then primary antibodies in blocking buffer at 4 °C overnight. The samples were washed and incubated with secondary antibodies for 1.5 h at room temperature. Images were acquired using a laser‐scanning confocal microscope (Nikon, Japan). The cross‐sectional area and minor axis ratio were evaluated by quantifying the NF200^+^ area (green) using imageJ software.

NMJ and muscle staining were done as described previously.^[^ ^26^ ^]^ Briefly, rats were deeply anesthetized with overdosed isoflurane and perfused with 0.01 M PBS followed by 4% paraformaldehyde (PFA). The extensor digitorum longus, the TA muscle, and the sciatic nerve was isolated and fixed in 4% PFA for 24 h. The extensor digitorum longus was divided into small bundles under a stereomicroscope. The samples were washed four times with 0.01 M PBS at 10‐min intervals, treated with 0.1 M glycine for 45 min, and washed four more times with 0.01 M PBS at 10‐min intervals. They were then permeabilized with 2% Triton‐100/PBS over 48 h, blocked with 5% BSA for 120 min, and incubated with primary antibodies over 16 h at 4 °C. The samples were washed and incubated with the appropriate secondary antibodies for 1.5 h at room temperature. The NMJ innervation ratio was determined by NF‐L&SYN^+^ NMJs / total NMJs (α‐BTX^+^).

For muscle cross‐section staining, the TA muscles were dehydrated in 30% sucrose at 4 °C for 48 h. The transverse sections (12 µm) were taken using a cryostat microtome (CM1950, Leica, Germany). Then, the sections were rinsed with PBS and incubated with blocking buffer (1% Triton X‐100 in 3% BSA and 2% goat serum) for 2 h at room temperature and then primary antibodies in blocking buffer at 4 °C overnight. The samples were washed and incubated with secondary antibodies for 1.5 h at room temperature. The type 2 and type 2BX myosin were determined by MyHC‐1^+^ (blue) and unstaining fibers, respectively.

### Hematoxylin‐Eosin (H&E) Staining and Evaluation of the Sciatic Nerves

Rat sciatic nerves were fixed in 10% PFA for at least 36 h, embedded in paraffin, sectioned, and stained with H&E. Images were taken by DM4 B microscope (Leica, Germany). Immune infiltration score was evaluated according to previous literature as 0, 1, 2, 3, and 4, indicating 0%, 25%, 50%, 75%, and >75% infiltration, respectively.^[^ ^27^ ^]^

### Histological Staining of Muscle

After sacrificing the rats, TA were isolated, immersed in 10% PFA for 36 h, and subsequently embedded in paraffin. Slices (5 µm) of the maximum cross section of the muscle were obtained. The evaluation of collagen deposition was performed using Masson trichrome staining, while muscle fiber area was assessed through HE staining.

### Transmission Electron Microscopy Evaluations

After perfusion of the mSNI rats, a 5 mm segment of the sciatic nerve distal to the compression site was collected and fixed in 2.5% glutaraldehyde. The samples were then post‐fixed with 1% OsO4 solution for 1 h, washed, dehydrated using a gradient of alcohol concentrations, and embedded into Epon 812 epoxy resin. The samples were cut into ultrathin (60 nm) sections, stained with lead citrate and uranyl acetate, and observed by transmission electron microscopy (TEM, Hitach HT770, Japan). Images of six random fields were used to calculate the diameter of myelinated nerve fibers, G‐ratio, axon density, and the thickness of myelin sheaths.

### Muscle Contraction Evaluations

The mSNI rats were anesthetized and kept on a heated pad to maintain body temperature. The surface temperature was measured with an infrared thermometer every 30 min. The room temperature was maintained at 26 °C. The rats were subsequently positioned in the testing apparatus (701C stimulator, 300D‐305C force transducer, 806D test apparatus, Aurora Scientific, Canada). One TA muscle can be measured repeatedly in vivo by inserting one needle‐like stimulating electrode percutaneously into the belly of the TA muscle and another one into the common peroneal nerve (CPN) near the fibular head. Ankle movement torque, an indicator of TA muscle contraction, was measured with a pedal. Following two twitches (1 mA, 0.2 ms), the muscles were stimulated by a series of tetanic pulses (1 mA, 400 ms) at 20, 40, 50, 60, 80, and 100 Hz with an interval of 1 min. Subsequently, the muscles were rested for 2 min before being stimulated with three tetanic pulses (1 mA, 400 ms, 100 Hz) at 1‐min intervals on average.^[^ ^28^ ^]^

### SFI Evaluation

Before testing, each rat was conditioned to navigate a 90 cm long and 13 cm wide channel leading to a food‐containing box. Pristine paper was prepared to document their footprints. The rats with inked hind paws were guided through the channel, and a minimum of three consecutive sets of clear footprints were recorded. The SFI was computed utilizing the methodology delineated by Bain JR et al.^[^ ^29^ ^]^ All behavioral tests were conducted 12 h after the compression was increased, when the pressurization and behavioral test were arranged on the same day.

### dSFI Evaluation

DigiGait (Mouse Specifics, USA) imaging system was used for photographic gait acquisition and gait image analysis of mSNI rats. Before testing, the genital area was coated with a black safety dye to evade analysis. The rats were then placed on the treadmill with an initial speed of 10 cm^−1^s, which was gradually increased to 18 cm^−1^s. The test was stopped if a continuous gait lasting more than 6 s was recorded.^[^ ^30^ ^]^ The movements of the rats' limbs as they approached or left the treadmill were captured by a high‐speed digital camera. The images were automatically corrected and a variety of gait parameters including SFI were analyzed. The dSFI value was obtained using digigait software.

### Hargreaves Test

The mSNI rats were placed in a Hargreaves testing device (Xinruan XR1800, China) and allowed to acclimate for 20 min, during which the absence of feces at the bottom was confirmed. The soles of the affected limbs of mSNI rats were subjected to thermal stimulation, and the duration of stimulation required for leg raising response in the rats was recorded. The measurements were conducted three times at 10‐min intervals, and the average of these three measurements was utilized as the final outcome.

### Mechanical Pain Threshold Evaluation

To evaluate mechanical hyperpathia, the mSNI rats were placed on an elevated mesh rack and housed in individual chambers.^[^ ^31^ ^]^ The central plantar surface of affected hind paw was stimulated with a series of von Frey filaments. The 50% paw withdrawal threshold was calculated using an up‐down protocol.^[^ ^32^ ^]^

### Electromyography

The mSNI rats were anesthetized and placed on a warming device to maintain body temperature. Following hair removal on the right lower limb, an electrode was carefully inserted proximal to the compression site without any potential damage to the sciatic nerve. The compound muscle action potentials (CMAPs) of the gastrocnemius were recorded using Keypoint4 electromyography system (Dantec, Denmark).

### Whole‐Cell Patch Clamp Recordings in Whole‐Mount DRGs Ex Vivo

Whole‐mount DRG patch clamp recordings were employed to examine nociceptor hypersensitivity.^[^ ^1c^ ^]^ General anesthesia was performed with isoflurane in oxygenized air provided by a small animal anesthesia device (RWD, China). Surgeries were performed under an operating microscope (Nikon, Japan). L4‐6 DRGs were isolated and placed in ice‐cold dissection solution oxygenated by 95% O_2_ and 5% CO_2_. After dissecting away the connective tissue, intact DRGs were mildly digested by 0.4 mL collagenase A (1 mg mL⁻^1^) and trypsin (0.25%) for 20 min. Intact DRGs were then transferred to the recording chamber that was continuously perfused with oxygenated artificial cerebrospinal fluid (ACSF, 3 mL/min). Small‐diameter neurons (<25 µm) were identified by a 40 × water‐immersion objective (Olympus LUMPLFLN 40x, 0.8 N.A.) on a differential interference contrast microscopy (Scientifica). Whole‐cell patch clamp was made using borosilicate pipettes with resistance of 4–5 MΩ. Signals were acquired and processed by HEKA EPC 10 amplifier. Data acquisitions were performed with Patchmaster software, filtered at 1 kHz and sampled at 10 kHz. In current clamp mode, the action potentials were evoked by injecting current steps from 0–180 pA with an increment of 20 pA in 600 ms. Rheobase was measured by injecting currents from 0 pA with an increment of 20 pA in 200 ms. Data were recorded and analyzed by Patch Master software. Solutions recipes (in mmol): dissection solution: 225 sucrose, 26 NaHCO_3_, 2.5 KCl, 1.25 NaH_2_PO_4_, 0.5 CaCl_2_, 7 MgSO_4_, 26 D‐Glucose, 5 L‐Ascorbic Acid, and 3 Sodium Pyruvate, adjusted to pH 7.4 with NaOH; ACSF: 114.5 NaCl, 2.5 KCl, 1.25 NaH_2_PO_4_, 26 NaHCO_3_, 10 D‐Glucose, 2 MgSO_4_, 3 Sodium Pyruvate, 5 L‐Ascorbic Acid, and 5 L‐Glutamine, adjusted to pH 7.4 with NaOH; pipette solution: 116.5 potassium gluconate, 10 KCl, 5 Mg‐ATP, 10 Na_2_‐phosphocreatine, 0.3 Na_2_‐GTP, 0.5 EGTA, and 10 HEPES, adjusted to pH 7.3 with KOH.

### Statistical Analysis

All data are reported as mean ± s.e.m. Data were analyzed by GraphPad Prism 9. For behavioral tests and current evoked action potentials, two‐way ANOVA with Bonferroni's post hoc test was used. For other data, one‐way ANOVA with Bonferroni's post hoc test was used. Differences were considered significant when *p* < 0.05. For all the statistics data, asterisks correspond to the following significance levels: ns = not significant, *p < 0.05, **P < 0.01, ***P < 0.001, and ****P < 0.0001.

## Conflict of Interest

X.L., W.W., and S.C. are inventors of CN Patent Application ZL 201910709478.4, ZL 202010727396.5, ZL 202110257070.5, and ZL 202210815021.3, which covers the mClamp, magnetic control unit and are assigned to Jilin university. The remaining authors declare no competing interests.

## Author Contributions

T.Y., X.L., and R.C. contributed equally to this work. C.S. conceived and shaped the project. T.Y., X.L., R.C., X.Z., W.L., W.W., W.Y., and X.Z. performed experiments. R.C. and C.S. contributed to the experimental design. Illustrations were created by T.Y. using BioRender. T.Y. prepared the manuscript under the guidance of Z.G. and C.S. with feedback from all authors.

## Supporting information



Supporting Information

Supporting Information Video 1

## Data Availability

The data that support the findings of this study are available from the corresponding author upon reasonable request.
