# Supplementary material for: Establishment of a Magnetically Controlled Scalable Nerve Injury Model
Source: Adv Sci (Weinh). 2024 Sep 17;11(41):2405265. doi: 10.1002/advs.202405265 (PMC11538664; doi:10.1002/advs.202405265)
Supplement: Supplementary file 1 — Supporting Information [file ADVS-11-2405265-s001.docx]

Supporting Information

**
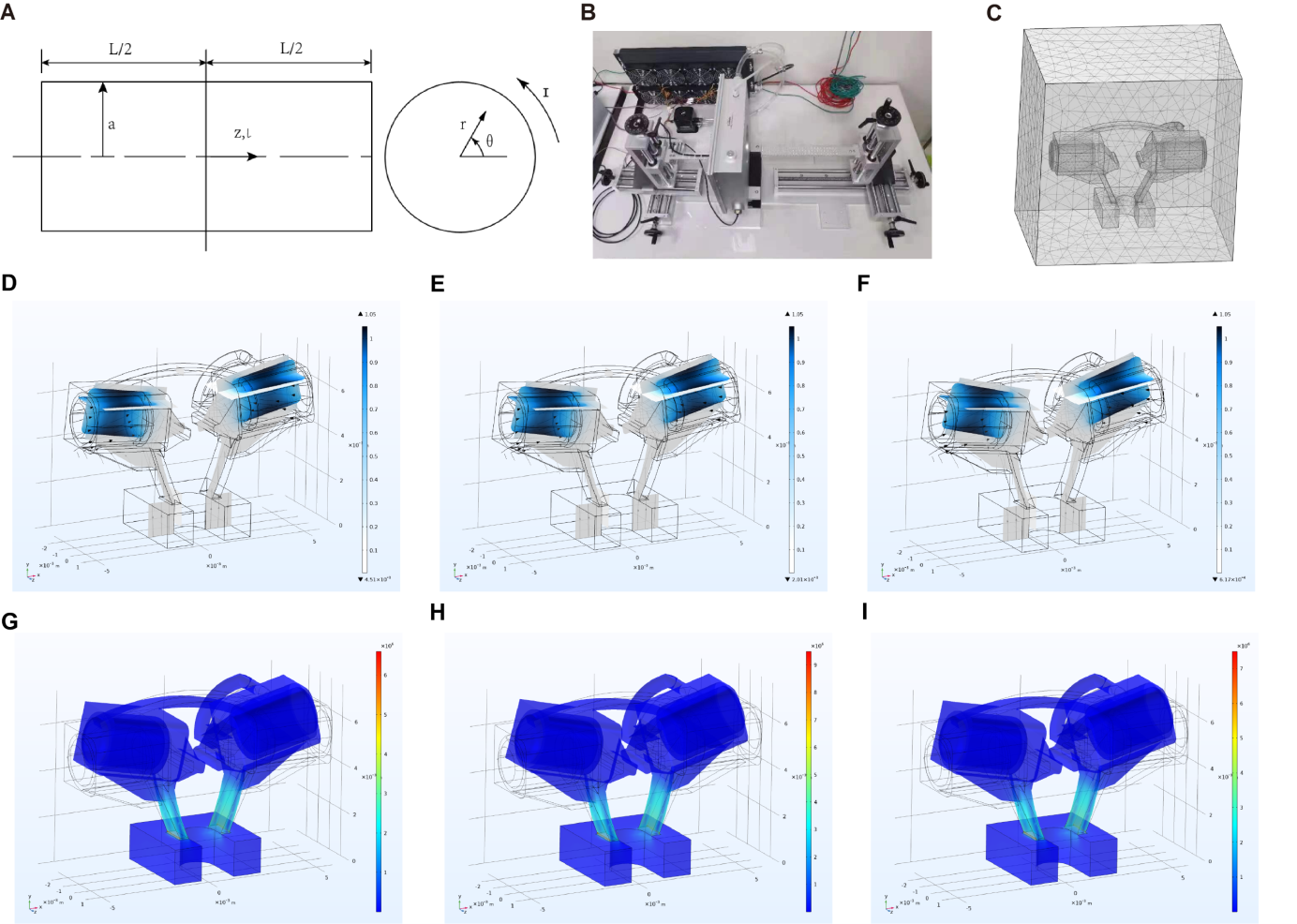
**

**Figure S1.** The magnetic control unit and finite element simulation.

A) Spiral coil coordinate illustration.

B) Profile display of the magnetic control unit.

C) Mesh of the finite element simulation.

D-F) Magnetic flux density distribution with 6 mT (D), 18 mT (E), and 36 mT (F) external magnetic field intensity.

G-I) Stress distribution on a 3D printed structure with external magnetic field of 6 mT (G), 18 mT (H), and 36 mT (I).

**
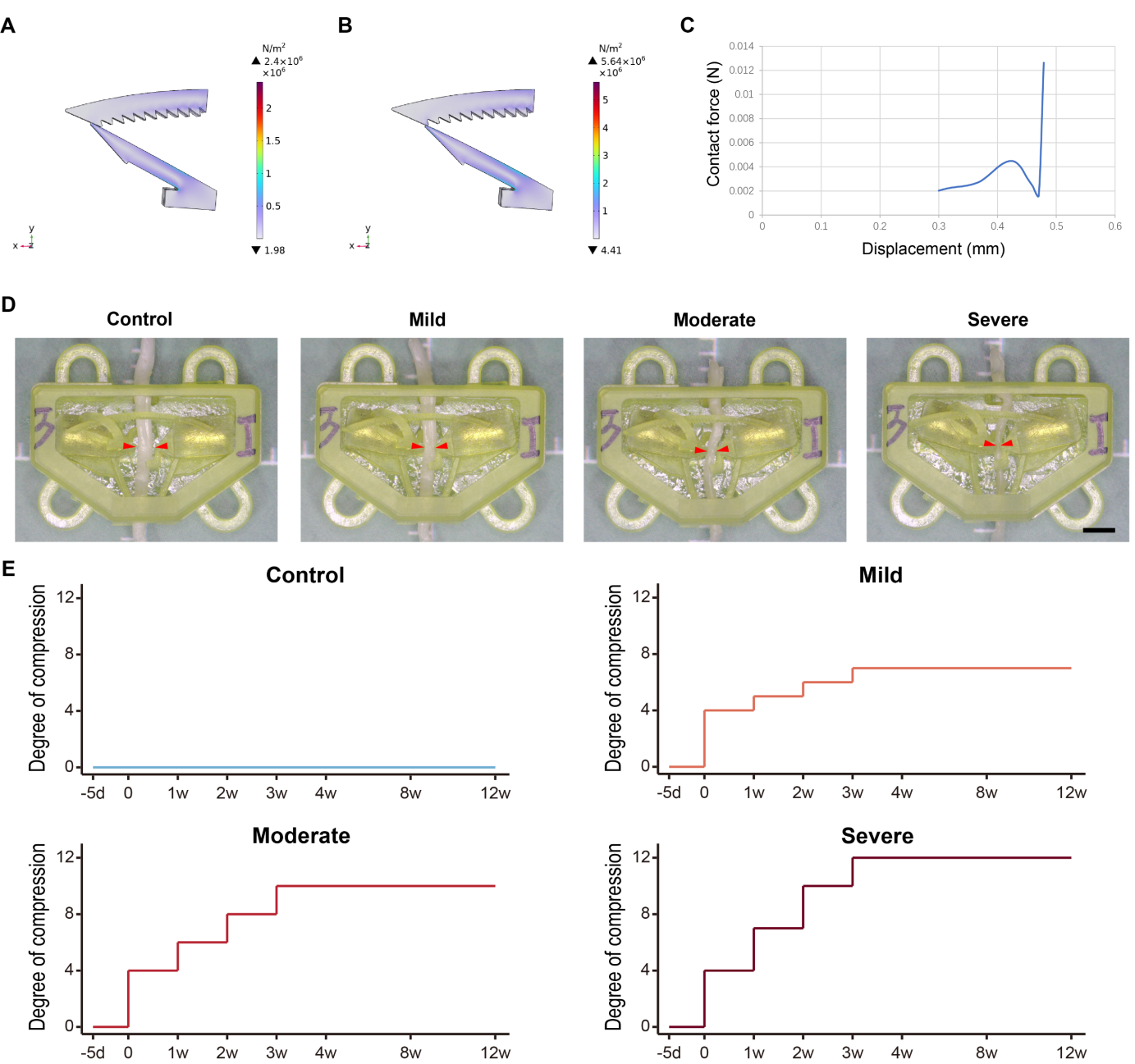
Figure S2.** Locking mechanism stress distribution and in vitro test of the mClamp.

A, B) Locking mechanism stress distribution at initial contact state (A) and final contact state (B).

C) Relationship between contact force and displacement.

D) In vitro test of the mClamp under condition of the final compression intensities of control, mild, moderate, and severe group. Compression points are indicated by red arrowheads. Scale bar is 2 mm.

E) Specific pressurization procedures for each group.

**
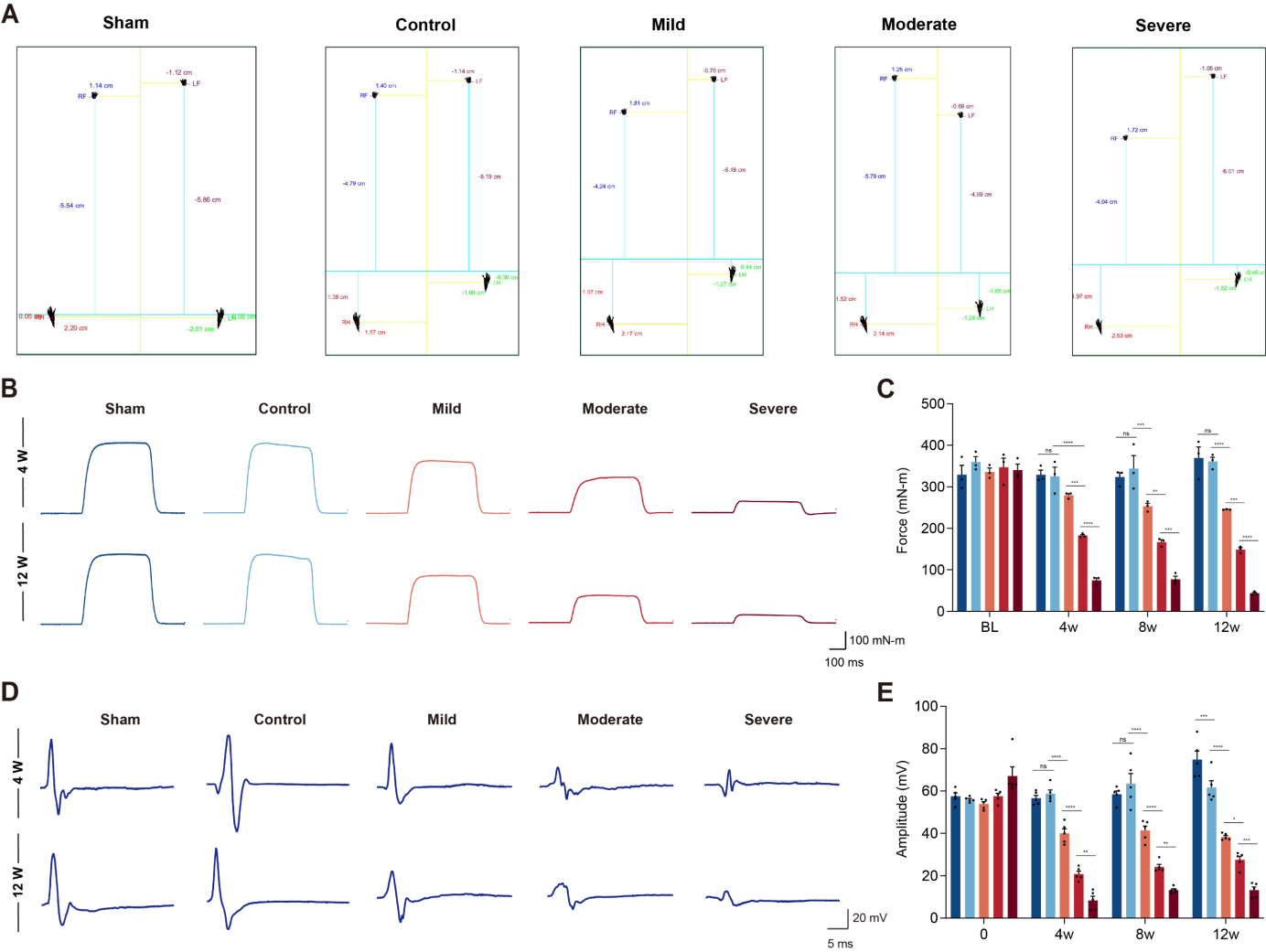
Figure S3.** dSFI and muscle-related evaluations.

A) Representative footprints recorded by the DigiGait system.

B, C) Representative traces (B) and quantification (C) of TA myodynamia. *n* = 3 rats per group.

D, E) Representative traces (D) and quantification (E) of TA electromyography. *n* = 5 rats per group. All data are expressed as the mean ± s.e.m. Statistical comparisons were conducted with two-way ANOVA followed by Bonferroni’s post hoc test.

**
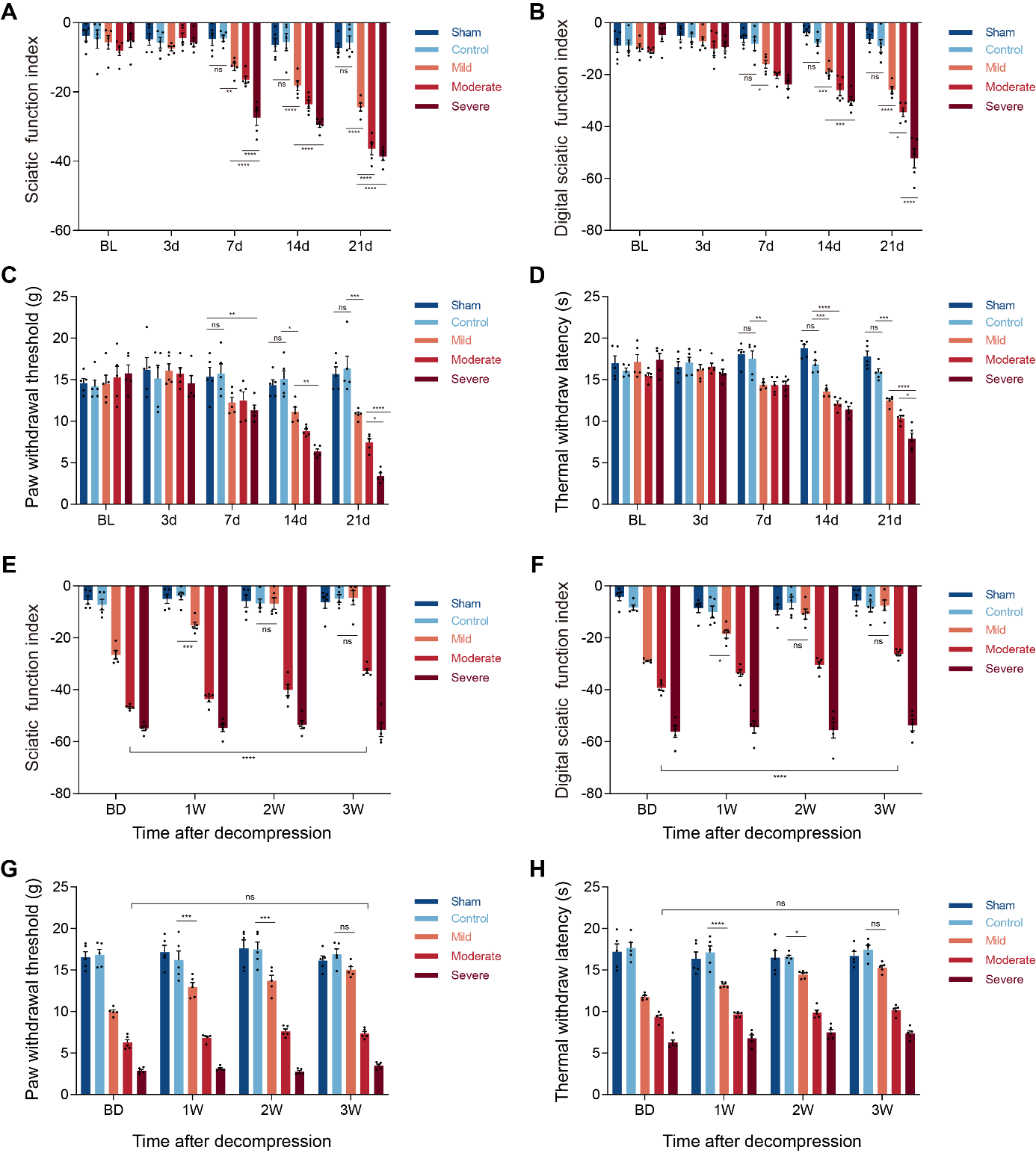
**

**Figure S4.** mSNI simulated the progression and recovery of chronic nerve entrapment syndromes.

A-D) The progression of neuropathic symptoms of mSNI rats at early stage, including motor function deficiency (A, B), mechanical (C) and thermal (D) pain hypersensitivity. *n* = 5 rats per group.

E-H) The recovery profile of mSNI rats after surgical decompression, including motor function deficiency (E, F), mechanical (G) and thermal (H) pain hypersensitivity. BD, before decompression. *n* = 5 rats per group.

All data are expressed as the mean ± s.e.m. Statistical comparisons were conducted with two-way ANOVA followed by Bonferroni’s post hoc test.

**
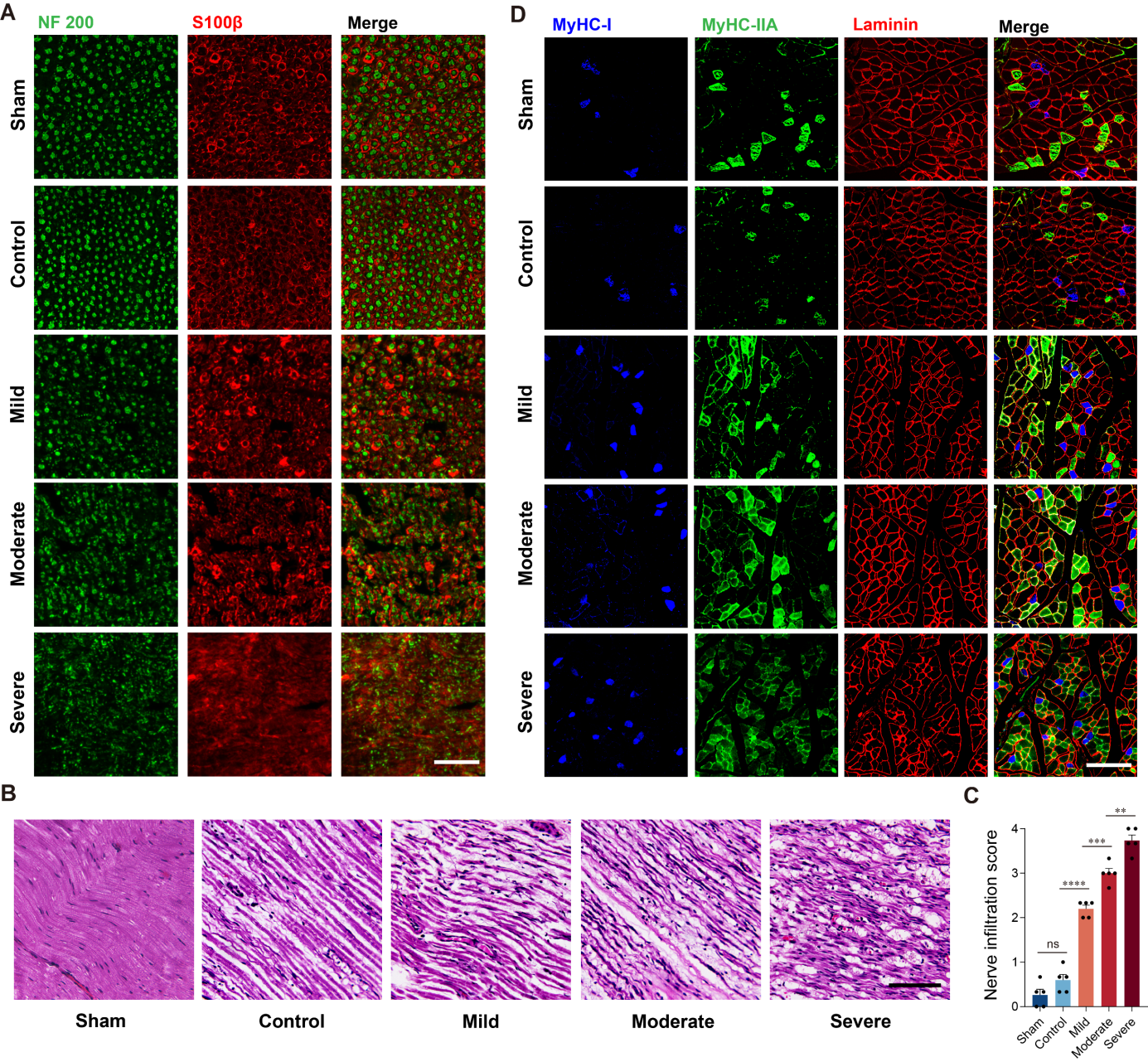
Figure S5.** Details of demyelination, H&E staining of the affected nerves and myosin of the affected muscles.

A) Details of immunostaining of the axon and myelin sheath of affected sciatic nerve 12 weeks after compression. Scale bar is 50 μm.

B, C) Representative images (B) and quantification (C) of H&E staining of the affected nerves at 4 weeks after compression began, 2mm proximal to the compression site. Scale bar is 100 μm. *n* = 5 rats per group.

D) Details of immunostaining of the myosin. Scale bar is 200 μm.

­­All data are expressed as the mean ± s.e.m. Statistical comparisons were conducted with one-way ANOVA followed by Bonferroni’s post hoc test.

**
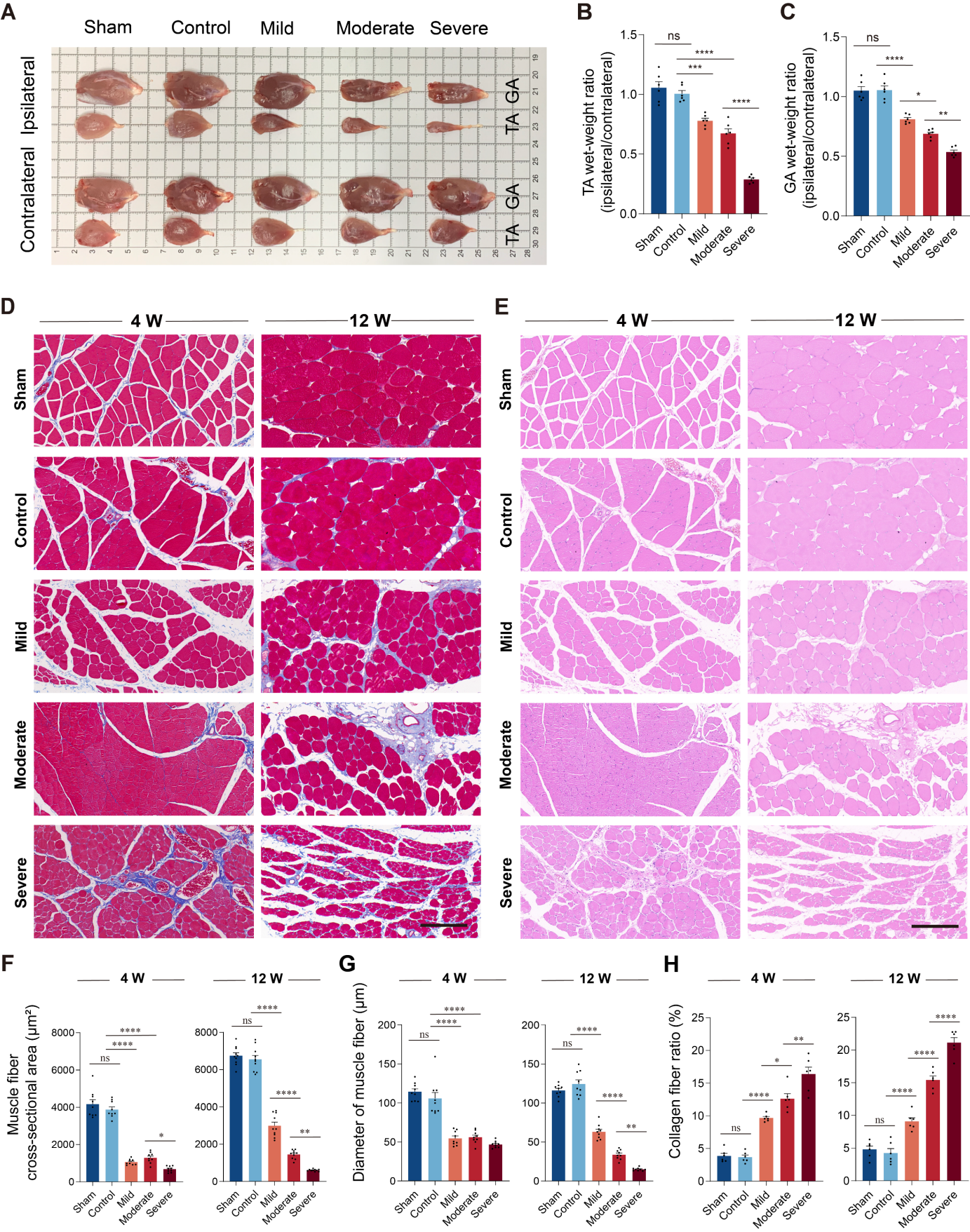
Figure S6.** Muscle weight and pathological staining.

A) Fresh TA and GA of contralateral and ipsilateral sides of mSNI rats. The unit of length is centimeter.

B-C) Wet-weight ratio of TA (B) and GA (C). *n* = 5 rats per group.

D, E) Masson (D) and H&E (E) staining of the affected anterior tibial muscle. Scale bar is 200 μm.

F-H) Quantification of muscle fiber cross-sectional area (F), diameter of muscle fiber (G), and collagen fiber ratio (H). *n* = 9 slices from 3 rats per group (F, G). *n* = 6 slices from 3 rats per group (H). All data are expressed as the mean ± s.e.m. Statistical comparisons were conducted with one-way ANOVA followed by Bonferroni’s post hoc test.

**
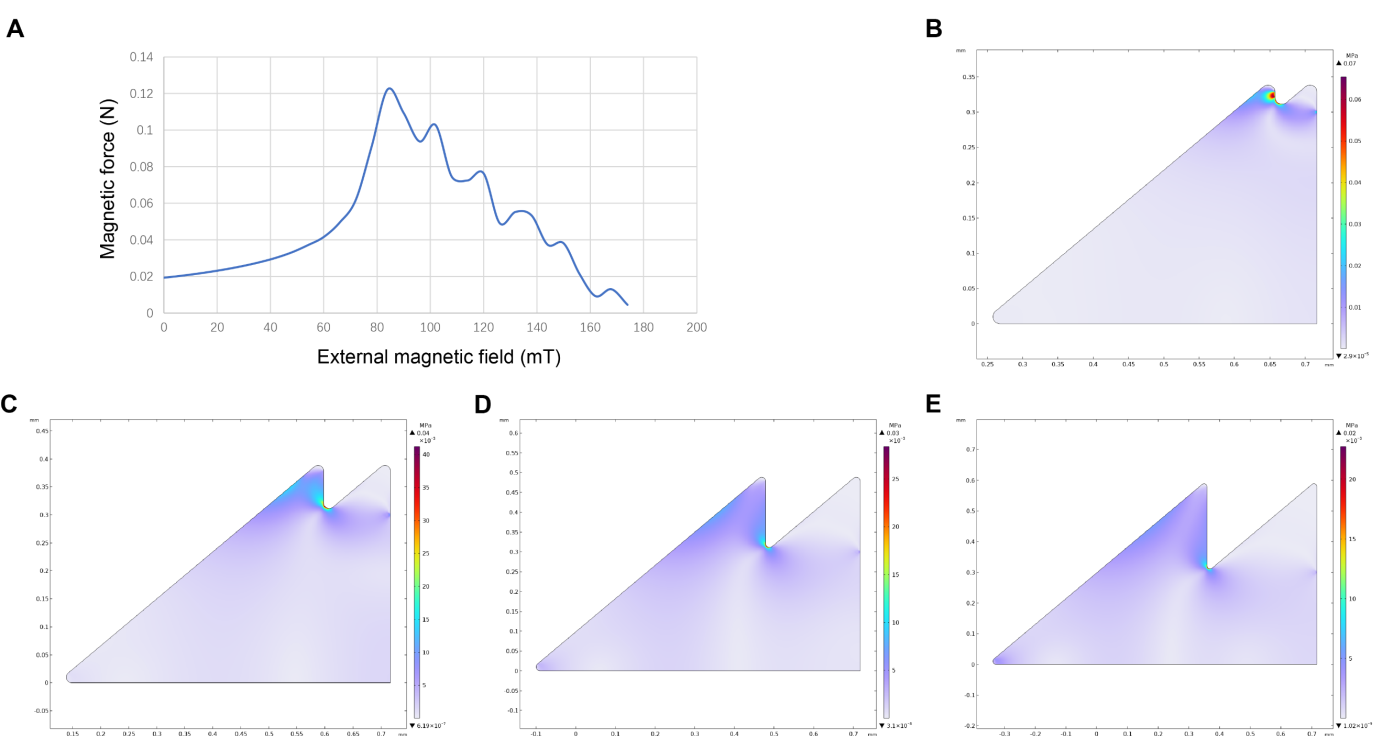
Figure S7.** The magnetic control unit and finite element simulation.

A) Relationship between the magnetic force of a permanent magnet and the external magnetic field.

B-E) Stress distribution for locking rack, tooth height 0.05 mm (B), 0.1 mm (C), 0.2 mm (D), and 0.3mm (E).

**Table S1. Mixtures details of the RG Resin**

| Name | Product identifier | % | Classification according to Regulation No. 1272/2008 [CLP] |
| --- | --- | --- | --- |
| Polymeric urethane acrylate | CAS-No.: 52404-33-8 | 25– 50 | Skin Corr./Irrit. 2 , H319 Eye Dam./Irrit. 2A , H315 |
| 7,7,9(or 7,9,9)-Trimethyl-4,13-dioxo-3,14- dioxa-5,12-diazahexadecane-1,16-diyl bismethacrylate | CAS-No.:72869-86-4 EC-No.: 276-957-5 | 25-50 | Skin Sens. 1B ,H317 Aquatic Acute 3 ,H402 Aquatic Chronic 2 , H411 |
| Oxydi-2,1-ethanediyl bismethacrylate | CAS-No.: 2358-84-1 EC-No.:219-099-9 | 20 – 25 | Skin Corr./Irrit. 3 , H316 Skin Sens. 1 , H317 Aquatic Acute 3 , H402 |
| Diphenyl(2,4,6-trimethylbenzoyl)phosphine oxide | CAS-No.: 75980-60-8 EC-No.: 278-355-8 | 1 – 3 | Skin Sens. 1B , H317 Repr. 2 (fertility) H361f Repr. 2 (unborn child) , H361 Aquatic Acute 2 , H401 Aquatic Chronic 2 , H411 |
| 2-Hydroxyethyl methacrylate | CAS Number: 868-77-9 EC-Number: 212-782-2 INDEX-Number: 607-124-00- X | 0-1 | Eye Dam./Irrit. 2B , H320 Skin Sens. 1B , H317 |

**Supporting Information**

**Design of NdFeb unit** The realization of our proposal involved the incorporation of NdFeB magnetic particles into a laser-curable resin. Due to its chemical reactivity and susceptibility to corrosion, the NdFeB material necessitates a specific surface coating to ensure adequate protection. In the absence of any protective coating, an oxide layer, > 100 μm in thickness, will be generated upon exposure to atmospheric conditions. A reduction in particle size increases the volume ratio of the surface oxide layer, thereby decreasing the proportion of NdFeB. The addition of NdFeB powder particles to the light-cured resin in 3D printing does not allow for the production of particles with a very small size. In addition, it is difficult to control the precise shape of NdFeB powder blend, due to the uneven UV light-curing process. Insufficient magnetic induction may result if the amount of the NdFeB powder is low. Thus, the final decision was made to adopt NdFeB magnetic beads embedded in the device.

**Correlation between the external magnetic field and induced clamping force** To examine the correlation between the intensity of the external magnetic field and the magnitude of the magnetic field force, the clamping force was oriented parallel to the x-axis. The magnetic field strength in the x-direction of a solitary permanent magnet when subjected to an external magnetic field was analyzed (Figure S7a), and we found that the induced force on the permanent magnet increased proportionally with the increase in the external magnetic field. Nevertheless, once the maximum value is surpassed, the angle between the permanent magnet and the external magnetic field undergoes a reversal, leading to a swift decline in the induction force. In practical terms, a reversal cannot take place without causing any structural failure, owing to the constraints inherent in the structure.

**Design of** **the locking mechanism** The spring support experiences a maximum stress of 12 MPa, which is below the yield stress of the material. The yield stress of the material is estimated to be approximately 30 MPa. The locking mechanism comprises a rack for locking and a tooth for securing. The inclined locking rack exhibits an angle of 40 degrees and has a specified height of 0.2 mm as per its design. The magnetic field simulation results were utilized to apply a magnetic field force to the locking rack. Subsequently, a model was developed to examine the forces acting on the locking rack. The height of the locking rack varied from 0.05 mm to 0.3 mm, and the stress distribution was analyzed (Figure S7b-e). The maximum effective stress observed in the structure was 0.02 MPa, indicating that it functions considerably below its failure stress. As a result, the forces on structure were deemed to be within a safe range. The study revealed that a decrease in tooth spacing could cause printing errors, ultimately leading to inadequate tooth depth. This, in turn, could result in disengagement of the locking teeth and subsequent failure to secure the mechanism. Thus, it can be concluded that the optimal size for the locking tooth was 0.2 mm.
